# Protective effects of low-intensity pulsed ultrasound on aluminum-induced cerebral damage in Alzheimer's disease rat model

**DOI:** 10.1038/srep09671

**Published:** 2015-04-15

**Authors:** Wei-Ting Lin, Ran-Chou Chen, Wen-Wei Lu, Shing-Hwa Liu, Feng-Yi Yang

**Affiliations:** 1 Department of Biomedical Imaging and Radiological Sciences, National Yang-Ming University, Taipei, Taiwan; 2 Biophotonics and Molecular Imaging Research Center, National Yang-Ming University, Taipei, Taiwan; 3 Institute of Toxicology, College of Medicine, National Taiwan University, Taipei, Taiwan; 4 Department of Radiology, Taipei City Hospital, Taipei, Taiwan

## Abstract

The protein expressions of neurotrophic factors can be enhanced by low-intensity pulsed ultrasound (LIPUS) stimulation in the brain. The purpose of this study was to demonstrate the protective effect of LIPUS stimulation against aluminum-induced cerebral damage in Alzheimer's disease rat model. LIPUS was administered 7 days before each aluminum chloride (AlCl_3_) administration, and concomitantly given with AlCl_3_ daily for a period of 6 weeks. Neurotrophic factors in hippocampus were measured by western blot analysis. Behavioral changes in the Morris water maze and elevated plus maze were examined in rats after administration of AlCl_3_. Various biochemical analyses were performed to evaluate the extent of brain damages. LIPUS is capable of prompting levels of brain-derived neurotrophic factor (BDNF), glial cell line-derived neurotrophic factor (GDNF), and vascular endothelial growth factor (VEGF) in rat brain. AlCl_3_ administration resulted in a significant increase in the aluminum concentration, acetylcholinesterase activity and beta-amyloid (Aβ) deposition in AlCl_3_ treated rats. LIPUS stimulation significantly attenuated aluminum concentration, acetylcholinesterase activity, Aβ deposition and karyopyknosis in AlCl_3_ treated rats. Furthermore, LIPUS significantly improved memory retention in AlCl_3_-induced memory impairment. These experimental results indicate that LIPUS has neuroprotective effects against AlCl_3_-induced cerebral damages and cognitive dysfunction.

Developing effective and safe treatment strategies for brain disorders, such as dementia, Alzheimer's disease (AD), and Parkinson's disease (PD), remains a challenge in the field of clinical application. AD is the most common form of dementia that has no cure. Environmental factors have been thought to be one of possible reasons for induction of brain disorders^1^. Aluminum (Al) is the most abundant metal in the earth's crust and it may enter the human body through food, drinking water, and Al-containing drugs^2^. Al exposure is known to be neurotoxic and can induce cognitive deficiency and dementia^3^,^4^. Although the connection between AD and Al still exist controversies, experimentally it has been demonstrated that chronic exposure to Al causes neuropathological changes and cognitive impairments which are similar to those of AD^5^. Al accelerates Aβ generation and increases the formation of beta-amyloid (Aβ) oligomers^6^,^7^. It has also been reported that chronic aluminum chloride (AlCl_3_) administration in rats showed significant increase in the brain acetylcholinesterase (AChE) activity as compared to control rats^8^,^9^. At the cellular and molecular levels, AD is characterized by the deficiency of the neurotransmitter acetylcholine, extracellular Aβ deposits, neurofibrillary tangles, and the loss of neurons^10^.

Currently AD is limited to chemotherapies that promote neurotransmitter function by AChE inhibitors^11^,^12^. Emerging treatment strategies focus on reducing Aβ plaques from the brain of AD patients^13^,^14^. Furthermore, the insufficient levels of endogenous neurotrophic factors may result in neurodegenerative diseases^15^. For instance, decreased expressions of brain-derived neurotrophic factor (BDNF) appear to play an important role in development of AD, Parkinson's disease (PD), and Huntington's disease^16^,^17^,^18^. Besides, vascular endothelial growth factor (VEGF) modulates axonal growth and new vessel formation^19^. It has been reported that increased levels of BDNF and glial cell line-derived neurotrophic factor (GDNF) has great potential for the treatment of AD and PD, respectively^20^,^21^. In general, clinical trials with neurotrophic factors face a major obstacle in how to circumvent the blood-brain barrier (BBB)^22^.

Focused ultrasound (FUS) with microbubbles holds great potential for targeted delivery of drugs to treat brain diseases after FUS-induced local BBB disruption^23^,^24^,^25^. Recently, one study demonstrated that FUS enhanced delivery of anti-Aβ antibodies rapidly reduces plaque in a model of AD with aggressive Aβ pathology^26^. It has also been shown that systemically administered neurotrophic factors can cross the disrupted BBB induced by FUS^27^,^28^. However, the systemic use of neurotrophic factors is hindered by short half-life in the systemic circulation and the risks involved with repeated injections. Additionally, the administration of exogenous BDNF and GDNF may cause side effects such as a pro-epileptic effect and cerebellar damage, respectively^29^,^30^.

Low-intensity pulsed ultrasound (LIPUS) is capable of stimulating intact brain circuitry and promoting levels of brain-derived neurotrophic factor (BDNF), an important regulator of long-term memory^31^,^32^. The results suggested the enhancement of endogenous neurotrophic factors via LIPUS could be a novel treatment strategy for brain disorder. Based on this background, present study was designed to investigate the neuroprotective effect of LIPUS against AlCl_3_-induced cognitive dysfunction and associated cerebral damage in rats.

## Results

### Ultrasound enhanced the expressions of neurotrophic factors in rat brain

**Figure 1:**
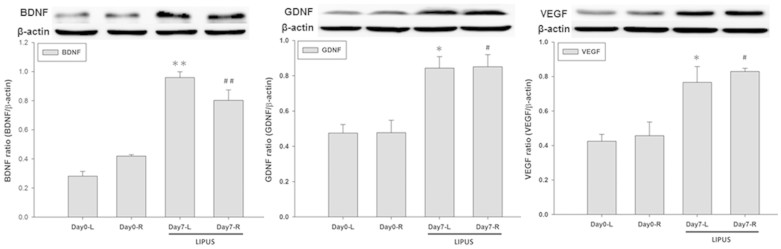
LIPUS-induced enhancement of neurotrophic factor expression in the rat brain. Each hemisphere was treated with LIPUS stimulations for a sonication time of 15 min. The hippocampus was subjected western blot to measure the protein expressions of BDNF, GDNF, and VEGF at 4 h following LIPUS stimulation. β–actin served as a loading control. The presented blots shown were derived from multiple gels. The membranes were cut based on molecular weights and probed with the antibody of interest. * and ^#^ denote significant differences in the sonicated hippocampi compared to the left and right ipsilateral control hippocampi, respectively. (*^,#^, *p* < 0.05, *n* = 4).

To investigate the effect of LIPUS on the protein levels of neurotrophic factors in the brain, bilateral rat hippocampi were exposed to multiple LIPUS stimulations for a sonication time of 15 min. The protein expressions of BDNF, GDNF, and VEGF in the stimulated hippocampi were significantly increased compared with the same expressions in the ipsilateral hippocampi of the control group (Fig. 1).

### Assessment of blood-brain barrier permeability

**Figure 2:**
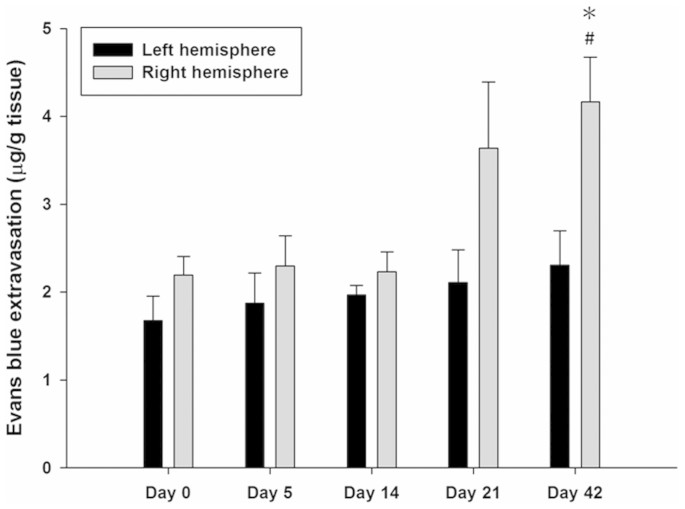
Graph showing the amount of Evans blue (EB) extravasation in left and right hemisphere as a function of day for AlCl_3_ administration in rats. EB extravasation was greatest on day 42 for the AlCl_3_-treated rats. * and ^#^ denote significant differences compared to the contralateral left hemisphere at the same time points and the right hemisphere at Day 0, respectively. (*^,#^, *p* < 0.05, *n* = 4).

The EB extravasation in the right hemisphere was significantly greater at 42-day exposure of Al compared to no exposure in Day 0 group, but no obvious difference was found at the other time points (Fig. 2). At day 42, EB extravasation in the right hemisphere was significantly greater than in the left hemisphere. However, no significant differences were found in the left hemisphere at any time points.

### Estimation of aluminum concentration and acetylcholinesterase activity

**Figure 3:**
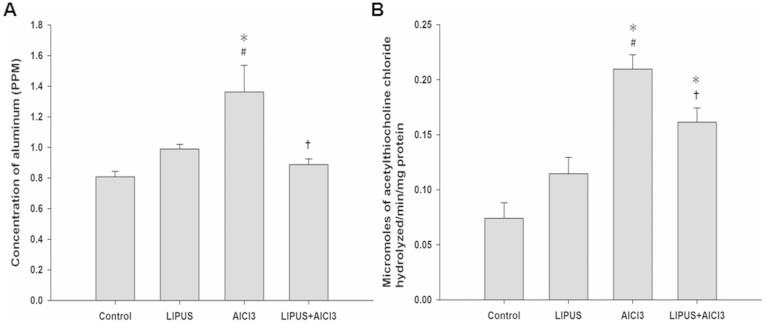
Effects of LIPUS on (A) aluminum levels and (B) acetylcholinesterase activity in AlCl_3_-treated rats. *, ^#^, and ^†^ denote significant differences compared to the individual groups of control, LIPUS, and AlCl_3_, respectively. (*^,#,†^, *p* < 0.05, *n* = 4).

AlCl_3_-treated rats showed significant increase in the aluminum concentration and AChE activity as compared to control. Chronic LIPUS stimulations in AlCl_3_-treated rats significantly attenuated the increase in aluminum concentration and AChE activity as compared to the AlCl_3_-treated rats (Fig. 3A and B). However, no significant differences were found in aluminum concentration and AChE activity after LIPUS stimulation in normal rats as compared to control.

### Aβ1-40 and Aβ1-42 protein levels

**Figure 4:**
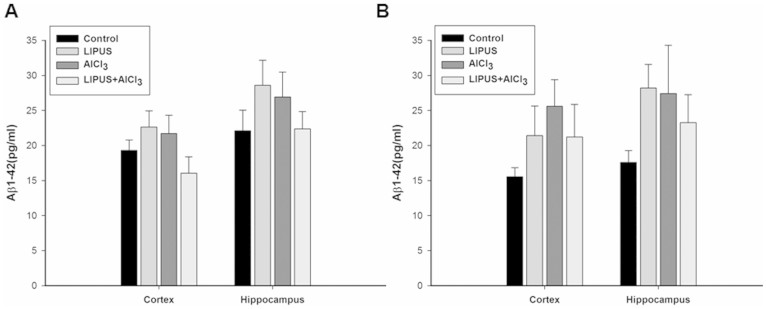
Effects of LIPUS on changes of Aβ content in cortex and hippocampus of brain induced by AlCl_3_ overload in rats. *, ^#^, and ^†^ denote significant differences compared to the individual groups of control, LIPUS, and AlCl_3_, respectively. (*^,#,†^, *p* < 0.05, *n* = 4).

There was no significant difference in the Aβ1-40 content of the cortex or hippocampus among individual groups (Fig. 4A). In contrast, a significant increase in Aβ1-42 expression was observed in the cortex and hippocampus of AlCl_3_-treated rats compared to control group (Fig. 4B). Chronic LIPUS stimulations in AlCl_3_-treated rats significantly attenuated the increase in Aβ1-42 expression as compared to the AlCl_3_-treated rats. Furthermore, no significant differences were found in Aβ1-42 expression for normal rats after LIPUS stimulation as compared to control group.

### Effect of LIPUS on memory performance

**Figure 5:**
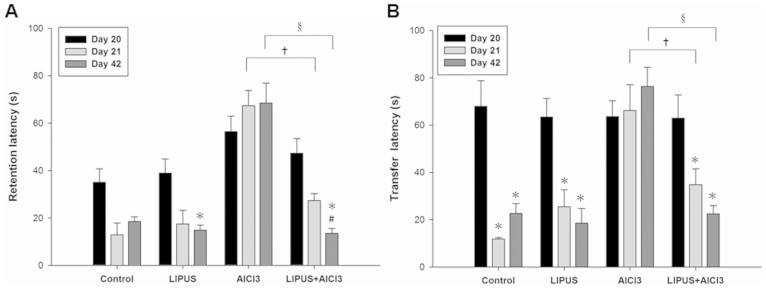
Effects of LIPUS on behavioral outcomes in AlCl_3_-treated rats. (A) Evaluation of ultrasound on memory retention in rats through Morris water maze test. The acquisition latency (AL) on day 20 and retention latency (RL) on days 21 and 42 in AlCl_3_-treated rats with or without LIPUS stimulation were observed. (B) Evaluation of ultrasound on memory performance in rats through elevated plus maze test. The transfer latency (TL) on days 20, 21, and 42 in AlCl_3_-treated rats with or without LIPUS stimulation were observed. * and ^#^ denote significant differences compared to the individual groups of latency on days 20 and 21, respectively. † and ^§^ denote significant differences between AlCl_3_-treated group with and without LIPUS stimulation on days 21 and 42, respectively. (*^,#,†,§^, *p* < 0.05, *n* = 6).

Rats treated only with AlCl_3_ showed learning and memory deficits in the Morris water maze task compared to control group rats (Fig. 5A). There was an increase in the mean acquisition latency (AL) of the AlCl_3_-treated group when compared to the control group on day 20. By contrast, a combination treatment of LIPUS and AlCl_3_ resulted in a mildly decreased AL as compared to rats treated only with AlCl_3_ on day 20. Following training, the mean retention latency (RL) was decreased in the control group on days 21 and 42, respectively, as compared to the AL on day 20. The LIPUS treatment of AlCl_3_-treated rats resulted in a significant decline in RL on days 21 and 42, respectively, as compared to the RL in rats treated only with AlCl_3_. Moreover, a significant decrease in RL on day 42 was found in LIPUS+ AlCl_3_ group as compared to the AL on day 20. These results suggest that the retention performance for the spatial navigation task was improved by LIPUS stimulation.

In the elevated plus maze, memory was evaluated and termed as transfer latency (TL). On day 20, mean TL for each group was relatively stable and showed no significant difference (Fig. 5B). Following training, mean TL in control and LIPUS groups on days 21 and 42 were significantly decreased as compared to TL on day 20, respectively. In contrast, no significant differences were found in the mean TL of AlCl_3_ treated group on days 21 and 42 as compared to pre-training TL on day 20. There was a significant increase in the mean TL of AlCl_3_ treated group when compared to control group on days 21 and 42. However, there was no statistical change in the combination of LIPUS and AlCl_3_ offered treated group as compared to control group on days 21 and 42. Furthermore, the LIPUS treatment of AlCl_3_-treated rats resulted in a significant decline in TL on days 21 and 42, respectively, as compared to the TL in rats treated only with AlCl_3_. The LIPUS stimulation alleviated the AlCl_3_-induced learning and memory deficits in rats.

### Histological observation

**Figure 6:**
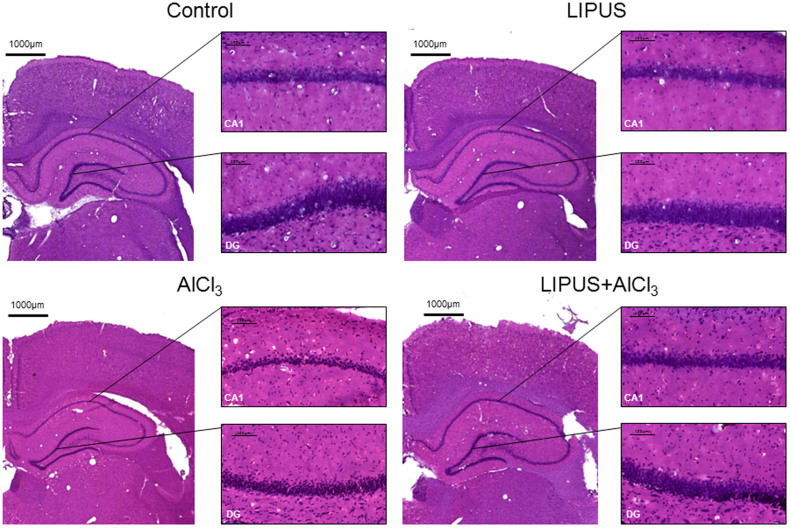
Effects of LIPUS on cerebral damage to hippocampus (CA1) and dentate gyrus (DG) in AlCl_3_-treated rats. H&E staining revealed that LIPUS stimulation on AlCl_3_-treated rats had fewer karyopyknosis of cells than AlCl_3_-treated rats. The scale bar is 100 μm in amplified regions.

As shown in Figure 6, karyopyknosis (shrinkage of cell nuclei) was observed in the hippocampal CA1 and dentate gyrus (DG) of AlCl_3_-treated rats with or without LIPUS stimulation. Furthermore, fewer cells with karyopyknosis were found in AlCl_3_-treated rats with LIPUS stimulation compared with the AlCl_3_ group. The LIPUS treatment ameliorates the cerebral damage in the AlCl_3_-treated rats.

## Discussion

It has been shown that an association exists between chronic exposure to Al in drinking water and AD risk^33^,^34^. Furthermore, chronic exposure of Al increased AChE activity and Aβ deposition in vivo studies^8^,^35^. The present study revealed that AlCl_3_ could enter the brain in rats and enhance the AChE activity and Aβ1-42 content as well as cause brain damage, such as karyopyknosis. Chronic LIPUS stimulation was able to improve cognitive dysfunction and ameliorate the cerebral damage, suggesting its potential role as a neuroprotectant against Al-induced neurotoxicity.

FUS-induced BBB disruption is not limited to improving delivery of therapeutic agents to the brain tumor^36^,^37^. This technology has potential applications in the treatment of neurodegenerative diseases, such as improving access of exogenous neurotrophic factors to normal brain and delivering antibodies for AD brain in mice^26^,^27^,^38^. However, several issues need to be further resolved to ensure the treatment efficacy before clinical applications. Regarding the enhancement of delivery, a recent published report has shown that not all neurotrophic factors will successfully cross the BBB after FUS-induced BBB disruption^27^. The discrepancy may lie in the fact that significant differences in the systemic circulation and various amount of receptors in the brain for neurotrophic factors. Besides, the safety of BBB disruption by FUS in AD models should be carefully evaluated. Although drug delivery to the brain in AD could be improved by FUS, the optimal ultrasound parameters for BBB disruption are hard to decide due to the status of the BBB in AD is variable between patients. Our data indicated that BBB disruption is dependent on exposure of Al to the rat brain (Fig. 2). Moreover, significant differences in the degree of BBB disruption were found between left and right hemispheres after chronic AlCl_3_ administration. Based on the variable disruption of BBB, side effects involved with high dosage levels are difficult to avoid because the enhancement of exogenous neurotrophic factors in AD brain by FUS is not always to be predictable.

LIPUS can stimulate electrical activity in neurons by activating sodium- and calcium-gated channels^39^. Additionally, LIPUS induced a significant increase in the levels of BDNF in hippocampus^31^. Similarly, the LIPUS intensity (I_SPTA_ = 528 mW/cm^2^) we found sufficient for promoting neurotrophic factors is below the ultrasound intensity limits (I_SPTA_ < 720 mW/cm^2^) set by the United States Food and Drug Administration for diagnostic imaging. Our data demonstrated that LIPUS can noninvasively promote the levels of neurotrophic factors in the rat hippocampus (Fig. 1). Significantly improved memory retention and attenuated AChE activity levels, and Aβ deposition produced by LIPUS lend support to our hypothesis that transcranial LIPUS has a neuroprotective effect against Al-induced cerebral damage (Fig. 3B, Fig. 4B and Fig. 5). Besides, the clearance of potential neurotoxins from the brain would be decreased due to neurovascular reductions in neurodegenerative diseases^40^,^41^. For example, the length of brain capillaries is reduced in AD. As shown in Fig. 1, the levels of VEGF can be significantly elevated by LIPUS in the brain. VEGF is a sub-family of growth factor that stimulates vasculogenesis and angiogenesis. This might explain why the Aβ content significantly decreased in AlCl_3_-treated rats after chronic LIPUS application (Fig. 4B).

Before translation to clinical applications, the biomechanical mechanisms and treatment efficacy still need to be assessed for LIPUS deep-brain stimulation. Ongoing work at our groups is aimed at understanding the signaling pathway to LIPUS stimulation and demonstrating the neuroprotective effects of chronic LIPUS treatment for transgenic AD models. In conclusion, we are the first to show that chronic LIPUS stimulation significantly prevented Al overload-induced damage of learning and memory function, karyopyknosis, inhibited rising of AChE activity, and down-regulated the protein expression of Aβ content. The use of LIPUS could potentially increase neurotrophic factors and perhaps aid with controlling or reversing related brain disorders such as AD.

## Methods

### Animal preparation

**Figure 7:**
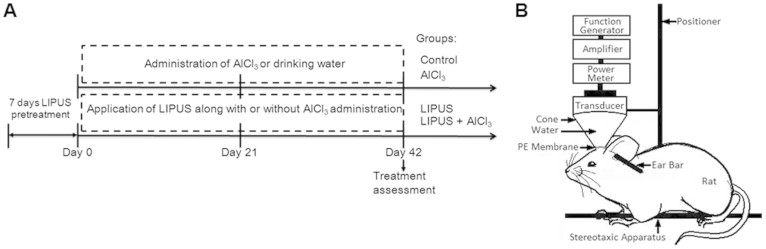
Experimental design. (A) Flow chart of the experimental procedure. (B) Schematic diagram of low-intensity pulsed ultrasound setup.

All procedures involving animals were in accordance with the guidelines for the Care and Use of Laboratory Animals. This study protocol was approved by Animal Care and Use Committee of National Yang Ming University. Male Sprague-Dawley (SD) rats weighing from 160 to 170 g were used in this study. Before LIPUS stimulation, each animal was anesthetized in the prone position by inhalation of 2% isoflurane in 2 l/min oxygen, and the body temperature was maintained at 37°C using a heating pad. The rat heads were mounted on a stereotaxic apparatus (Stoelting, Wood Dale, IL, USA), and the top of the cranium was shaved for LIPUS stimulation. For oral administration, AlCl_3_ was dissolved in drinking water. In one experimental protocol, the protein expression of neurotrophic factors on the normal rats treated with LIPUS daily for 0 and 7 days were evaluated by Western blotting. In another experimental protocol, animals were randomized into four groups (Control, LIPUS, AlCl_3_, and LIPUS+AlCl_3_) for biochemical analysis and behavioral assessment. The animals were treated with vehicle and served as control. Animals in group of LIPUS were treated with LIPUS daily for 49 days. In AlCl_3_ group, animals received AlCl_3_ (100 mg/kg; oral administration) daily for 42 days. The effects of LIPUS on the rats treated with AlCl_3_ (100 mg/kg) daily for 42 days were assessed in group of LIPUS+ AlCl_3_. LIPUS was applied daily for 49 days from 7 days before AlCl_3_ administration and lasted until the animals received AlCl_3_ daily for 42 days. All animals were sacrificed by decapitation following the day 42 after administration with AlCl_3_ (Fig. 7A).

### Pulsed ultrasound apparatus

LIPUS was generated by a 1-MHz focused piezoelectric transducer (A392S; Panametrics, Waltham, MA, USA) with 50 ms burst lengths at a 5% duty cycle and a repetition frequency of 1 Hz. The focused transducer was mounted on a removable cone filled with deionized and degassed water, the tip of which was capped by a polyurethane membrane, with the center of the focal zone placed about 5.7 mm away from the cone tip (Fig. 7B). The focused transducer was positioned using the stereotaxic apparatus in order to direct the acoustic beam to the desired region (3.0 mm posterior and 2.5 mm lateral to the bregma) of the brain. A function generator (33220A, Agilent Inc., Palo Alto, USA) was connected to a power amplifier (500-009, Advanced Surgical Systems, Tucson, AZ) to create the US excitation signal. A power meter/sensor module (Bird 4421, Ohio, USA) was used to measure the input electrical power. The spatial-peak temporal-average intensity (I_SPTA_) over the focused transducer head was 528 mW/cm^2^, and was measured with a radiation force balance (RFB, Precision Acoustics, Dorset, UK) in degassed water. LIPUS was transmitted from the top of the rat brain. US transmission gel (Pharmaceutical Innovations, Newark, NJ, USA) was used to cover the area between the transducer and the brain in order to maximize the transmission of the LIPUS. Each rat hemisphere was treated by LIPUS with triple sonications. The duration of each sonicaton was 5 min and there was an interval of 5 min between each sonication.

### Western blotting analysis

All rats were sacrificed 4 h after the last LIPUS stimulation. Fresh brain tissue in the hippocampus was homogenized by T-Per extraction reagent supplemented with the Halt Protease Inhibitor Cocktail (Pierce Biotechnology, Inc.). Lysates were centrifuged and the supernatants were harvested, and protein concentrations were assayed with Protein Assay Reagent (Bio-Rad, CA, USA). Samples containing 30 μg protein were resolved on 12% sodium dodecyl sulfate polyacrylamide gel electrophoresis (SDS-PAGE) and transferred to Immun-Blot® polyvinyldifluoride (PVDF) membranes (Bio-Rad, CA, USA). After blotting, the membranes were blocked for at least 1 h in blocking buffer (Hycell, Taipei, Taiwan), and then the blots were incubated overnight at 4°C in a solution with antibodies raised in rabbit against BDNF (1:250, sc-546, Santa Cruz, CA, USA), GDNF (1:250, sc-328, Santa Cruz, CA, USA), and VEGF (1:250, sc-152, Santa Cruz, CA, USA). After being washed with PBST buffer, the membrane was incubated with the secondary antibodies for 1 h at room temperature. After being washed with PBST buffer, signals were developed using a Western Lightning ECL reagent Pro (Bio-Rad, California, USA). The gel image was captured using ImageQuant™ LAS 4000 biomolecular imager (GE Healthcare Life Sciences, Pennsylvania, USA) and analyzed using Image J software (Image J, National Institute of Health, Bethesda, MD, USA) to estimate the integral optical density of the protein bands.

### Assessment of blood-brain barrier permeability

It has been shown that FUS-induced BBB disruption can be quantified based on the extravasation of EB^42^. Here, we used EB to evaluate the relationship between the dose of AlCl_3_ administration and BBB integrity. The animals were injected intravenously with EB (Sigma, St. Louis, MO) at a concentration of 100 mg/kg on the desired day (0, 5, 14, 21 or 42). The animals were sacrificed approximately 4 hours after the EB injection. Animals were perfused with saline via the left ventricle until colorless perfusion fluid appeared from the right atrium. After perfusion and brain removal, the brain was sectioned into three slices from 0 to 6 mm posterior to the bregma and these were mounted on glass slides. The coronal sections were then divided into right and left hemispheres before measuring the amount of EB extravasated. The group of Day 0 was used as the control group. Samples were weighed and then soaked in 50% trichloroacetic acid solution. After homogenization and centrifugation, the extracted dye was diluted with ethanol (1:3), and the amount of dye was measured using a spectrophotometer (PowerWave 340, BioTek, USA) at 620 nm. The content of EB in the tissue was quantified using a linear regression standard curve derived from seven concentrations of the dye and was denoted per gram of tissue.

### Aluminum concentrations in the brain

The aluminum was analyzed by wet acid digestion method^43^. The cortex and hippocampus of the brain were digested using a microwave. Measurement for Al concentrations in brain tissues were performed using furnace atomic absorption spectrometry (NexION 300 Series, Perkin Elmer, Waltham, MA, USA). The total concentration of Al was calculated in ng/mg of tissue.

### Acetylcholinesterase activity in the brain

AchE is a marker of extensive loss of cholinergic neurons in the forebrain. The activity of AChE was determined according to the Ellman method. The assay mixture contained 1 μl of supernatant, 180 μl of sodium phosphate buffer (pH 8), 9 μl of acetylthiocholine chloride and 10 μl of DTNB (Ellman reagent). The change in absorbance was measured for 2 min at 30 s interval at 412 nm using a spectrophotometer (Infinite® 200 PRO, Tecan, Switzerland). Results were expressed as micromoles of acetylthiocholine chloride hydrolyzed per min per mg protein.

### Aβ protein quantification with ELISA

On the 42nd day of the study, ELISA assessment was performed to evaluate the accumulation of Aβ in cortex and hippocampus of brain. The isolated tissue was homogenized in T-Per extraction reagent (Pierce Biotechnology, Inc., Rockford, IL) supplemented with the Halt Protease Inhibitor Cocktail (Pierce Biotechnology, Inc.). After centrifuging, supernatants were quantitated by amyloid beta ELISA kit (Uscn Life Science, Inc., Wuhan, China) according to the manufacturer's protocol. Briefly, protein samples were incubated with the first Aβ antibody pre-coated on plates for 1 h at 37°C. Then, secondary antibody was incubated for 30 min at 37°C. Finally, colorimetric reaction was conducted and absorbance on a spectrophotometer (Infinite® 200 PRO, Tecan, Switzerland) at 450 nm was recorded. The amount of amyloid beta proteins in tissue were quantified by a linear regression standard curve and were denoted picogram per ml.

### Behavioral assessment

In an in vivo behavioral experiment, 24 SD rats were randomized into four groups (Control, LIPUS, AlCl_3_, and LIPUS+AlCl_3_), each with 6 animals. The animals were challenged with AlCl3 (100 mg/kg) daily for the last 42 days to induce learning deficits and amnesia.

The acquisition and retention of a spatial navigation task was assessed by Morris water maze^44^. The pool is a custom-made black (200 cm × 60 cm) filled with water (23 ± 2°C). Opaque curtains surrounded the maze and were affixed with high-contrast visual cues (an X, a triangle, a circle, and a square). The pool was divided arbitrarily into four equally-sized quadrants (called zones I, II, III, and IV). The escape platform is a custom-made, clear plastic stand with a circular top measuring 20 cm in diameter. It sits approximately 2 cm above the surface of the water during the acquisition phase. The rats received a training session consisting of four trials on day 20 from the start of AlCl_3_ administration. The start locations were varied from trial to trial, with the rats being gently placed in the water facing towards the wall of the pool. The maximum swim time for the acquisition trial was 90s, after which the rat was guided to the platform and remained there for 20s following escape. The time spent by the rat to reach the platform was recorded and termed as AL. After completing the training trial, the rats were returned to the home cages and a 5 min gap was timed between the subsequent trials. Then, a similar platform was placed in the pool 2 cm below the water level for the maze retention phase. One day after the AL was recorded, the given rat was placed randomly at one of the edges facing the wall of the pool and tested for retention of response. The times spent to reach the platform on days 21 and 42 following the start of AlCl_3_ administration were measured and expressed as RL.

The elevated plus maze consisted of two open arms (50 cm × 12 cm), crossed with two closed walls raised 66 cm from floor level. Each rat was placed at one end of the open arm facing away from the center portion of the maze. The time spent by the rat to move from the open arm to the closed arm was measured as the TL on day 20 from the start of AlCl_3_ administration. The rats remaining in the open arm without entering into the closed arm within 90 s were pushed on the back into one of the enclosed arm and TL was recorded as 90 s. Similarly, retention of memory was evaluated as TL on days 21 and 42^45^.

### Histology

Three rats of each group were prepared for histological assessment. The rats were perfused with saline and 10% neutral buffered formalin at one day after the last LIPUS stimulation. The brains were removed, embedded in paraffin, and then serially sectioned into 30-μm-thick slices. The slices were stained with hematoxylin-eosin (H&E) to visualize their general cellular structure. Photomicrographs of 10 μm-thicknesses of the H&E-stained tissue sections were obtained using a Mirax Scan digital microscope slide scanner (Carl Zeiss, Mirax 3D Histech) with a Plan-Apochromatic 20/0.8 objective lens.

### Statistical analysis

All data are expressed as mean ± SEM. Statistical analysis was performed using one-way ANOVA. The behavioral assessment data were analyzed using the repeated measures two-way analysis of variance (ANOVA) followed by Tukey's test for multiple comparisons. The level of statistical significance was set at *p* value ≤ 0.05.

## Author Contributions

W.T.L., R.C.C., S.H.L. and F.Y.Y. designed the project, organized the entire research. W.T.L. and F.Y.Y. wrote the manuscript. W.W.L. performed the Western blotting analysis. All authors discussed the results and commented on the manuscript.
